# The microbiota-gut-brain-immune interface in the pathogenesis of neuroinflammatory diseases: a narrative review of the emerging literature

**DOI:** 10.3389/fimmu.2024.1365673

**Published:** 2024-05-16

**Authors:** Alison Warren, Yvonne Nyavor, Nikkia Zarabian, Aidan Mahoney, Leigh A. Frame

**Affiliations:** ^1^ The Frame-Corr Laboratory, Department of Clinical Research and Leadership, The George Washington University School of Medicine and Health Sciences, Washington, DC, United States; ^2^ Department of Biotechnology, Harrisburg University of Science and Technology, Harrisburg, PA, United States; ^3^ Undergraduate College, Princeton University, Princeton, NJ, United States

**Keywords:** human gastrointestinal microbiome, gut-brain axis, neuroimmunomodulation, enteric nervous system, neurogenic inflammation, neurodegenerative diseases, neuropathology, neuroinflammatory disease

## Abstract

**Importance:**

Research is beginning to elucidate the sophisticated mechanisms underlying the microbiota-gut-brain-immune interface, moving from primarily animal models to human studies. Findings support the dynamic relationships between the gut microbiota as an ecosystem (microbiome) within an ecosystem (host) and its intersection with the host immune and nervous systems. Adding this to the effects on epigenetic regulation of gene expression further complicates and strengthens the response. At the heart is inflammation, which manifests in a variety of pathologies including neurodegenerative diseases such as Alzheimer’s disease, Parkinson’s disease, and Multiple Sclerosis (MS).

**Observations:**

Generally, the research to date is limited and has focused on bacteria, likely due to the simplicity and cost-effectiveness of 16s rRNA sequencing, despite its lower resolution and inability to determine functional ability/alterations. However, this omits all other microbiota including fungi, viruses, and phages, which are emerging as key members of the human microbiome. Much of the research has been done in pre-clinical models and/or in small human studies in more developed parts of the world. The relationships observed are promising but cannot be considered reliable or generalizable at this time. Specifically, causal relationships cannot be determined currently. More research has been done in Alzheimer’s disease, followed by Parkinson’s disease, and then little in MS. The data for MS is encouraging despite this.

**Conclusions and relevance:**

While the research is still nascent, the microbiota-gut-brain-immune interface may be a missing link, which has hampered our progress on understanding, let alone preventing, managing, or putting into remission neurodegenerative diseases. Relationships must first be established in humans, as animal models have been shown to poorly translate to complex human physiology and environments, especially when investigating the human gut microbiome and its relationships where animal models are often overly simplistic. Only then can robust research be conducted in humans and using mechanistic model systems.

## 1 Introduction

While bacteria are the most commonly studied member, the gut microbiome consists of trillions of microbes including fungi, archaea, viruses, phages, and bacteria, which develop early in life and are influenced by genetic and environmental factors, including those that affect brain health (1). We cannot define what a ‘healthy’ gut microbiome is at present; however, low diversity is a common marker of an ‘unhealthy’ gut microbiome, which is often termed ‘dysbiosis.’ Dysbiosis is associated with many disease states, especially those becoming increasingly common in Western societies, likely due to limited exposure to diverse microbiota and inflammatory environmental exposures such as diet (2–4). This includes neuroinflammatory and neurodegenerative diseases, as dysbiosis contributes to gut and brain hyperpermeability, commonly termed ‘leaky gut’ and ‘leaky brain,’ by way of reduced tight junction proteins such as occludins (5). Microbial metabolites produced during dysbiosis are able to induce barrier dysfunction in preclinical models, leading to the passage of abnormal substances across barriers (6). The barrier function of the gut and the brain are an important part of innate immunity, without which the immune system cannot function properly, resulting in chronic inflammation locally and, perhaps eventually, systemically. This is a hallmark of dysregulation of the microbiota-gut-brain-immune interface. Moreover, dysbiosis can occur in any tissue containing a microbiome, including the oral and nasal cavities, lungs, skin, bladder, and vagina (7). While less studied than the gut microbiome, new evidence suggests the resident microbiota in these tissues can also contribute to immunoregulation and therefore a broad spectrum of disease states (7–9). Clues to the importance of microbiomes outside of the gut suggest some involvement in neuropsychiatric and neurodegenerative diseases, such as the presence of oral bacteria in the postmortem brains of persons with Alzheimer’s disease (7). Notably, the extent to which these localized microbiota contribute to disease is in an early stage of exploration; this paper will therefore focus on the more widely-studied gut microbiome. Accordingly, this narrative review will assess the state of the science in the emerging literature behind the microbiota-gut-brain-immune interface and the pathogenesis of neuroinflammatory diseases.

## 2 The microbiota-gut-brain-immune interface and neuroinflammation

Multidisciplinary research is emerging around the microbiota-gut-brain-immune interface, moving from animal models to human studies. This research is finding that the gut microbiota mediate the relationship between the enteric nervous system (ENS), autonomic nervous system (ANS), central nervous system (CNS) largely through regulation of the immune response and inflammation. The idea that brain function is tied to the gut microbiome and involves epigenetic and immunoregulatory changes is becoming common place in the clinic as well as in research. Further, nervous system epigenetic changes mediated by the gut microbiota show great promise to elucidate the pathogenesis of and novel therapeutics for neurological disorders (10). What is more, intimate and sophisticated relationships between diet, the gut microbiome, and cognition are emerging. Indeed, transdisciplinary perspectives intersecting neuroscience, psychology, and philosophy are further exploring the role of the gut microbiome in perception and cognition, and posit the idea that the microbiome possesses its own proto-cognition independent of, yet interrelated to the rest of the body (11). Interestingly, the hormones ghrelin and leptin (involved in hunger and satiety, respectively) have also been tied to cognition (12). Our microbiome, therefore, may not only affect the quality of our cognition but also how we perceive our internal and external worlds.

While external factors are important contributors to well-being and neuroinflammatory disease by way of epigenetic changes, internal factors (e.g. psyche, lifestyle, age, chronic inflammation, microbiomes) are at least equally important and interact with each other to potentiate a signal, perhaps synergistically. Changes to the epigenetics of the nervous system are typically acquired since neurons do not divide (13); the microbiota and their metabolites influence neurons (14–16). Microbiota-gut-brain-immune interface dysregulation has been associated with neuropathologies commonly linked with inflammation including mild cognitive impairment (MCI), Alzheimer’s disease, Parkinson’s disease, and multiple sclerosis and gastrointestinal (GI) symptoms are common in these disorders or even predate their onset (5, 17–23). For instance, GI symptoms predate the onset of Parkinson’s disease; this and a growing body of research support the theory that Parkinson’s starts in the gut and dysregulates the microbiota-gut-brain-immune interface, resulting in CNS and movement-related symptoms (21). GI symptoms may include nausea, constipation, dysphagia, abnormal salivation and defecatory dysfunction. Further, there is likely a bidirectional relationship, e.g. neuropsychiatric comorbidities are prevalent in inflammatory bowel disease (24). Thus, the microbiota-gut-brain-immune interface is a vital mediator of neuroinflammation likely to affect many facets of brain health including neurodevelopment, cognition, and behavior (20, 23). The most vulnerable aspects of the microbiota-gut-brain-immune interface to these effects involve multi-way physiological communication along the microbiota-gut-brain axis. Direct communication in the microbiota-gut-brain axis occurs predominantly via the vagus nerve while indirect signaling is diverse and complex including the ENS, ANS, CNS, immune system (e.g. glial activation), neuroendocrine system, tryptophan metabolism, and microbial metabolites (e.g. short-chain fatty acids, SCFAs) (18). Additionally, the gut microbiota are crucial to nutrient harvesting and produce some nutrients themselves that are co-factors for epigenetic pathways (25).

The spleen serves a crucial role in facilitating communication within the microbiota-gut-brain-immune interface by acting as a reservoir for various immune factors. Although the precise involvement of the spleen in the gut-brain axis is not completely understood, research indicates a correlation between antibiotic treatment and splenic function. Studies on mice subjected to antibiotic treatment have demonstrated a significant decrease in spleen weight, NK cells, macrophages, and neutrophils compared to control groups. Conversely, there is an observable increase in the percentage of CD8+ T cells within the spleen (26). Moreover, a proposed gut-spleen axis has been identified in patients with asplenia and common variable immune deficiency, wherein the reduction of IgM memory B cells induced by splenectomy may affect secretory IgA production in the gut. Numerous diseases, ranging from traumatic brain injury to conditions like Crohn’s disease, inflammatory bowel disease, septic shock, Alzheimer’s disease, Parkinson’s disease, schizophrenia, and depression, have been linked to the gut–brain–spleen axis. It is suggested that the vagus nerve reflex and systemic circulation serve as potential regulatory routes for these diseases (27).

**Figure 1:**
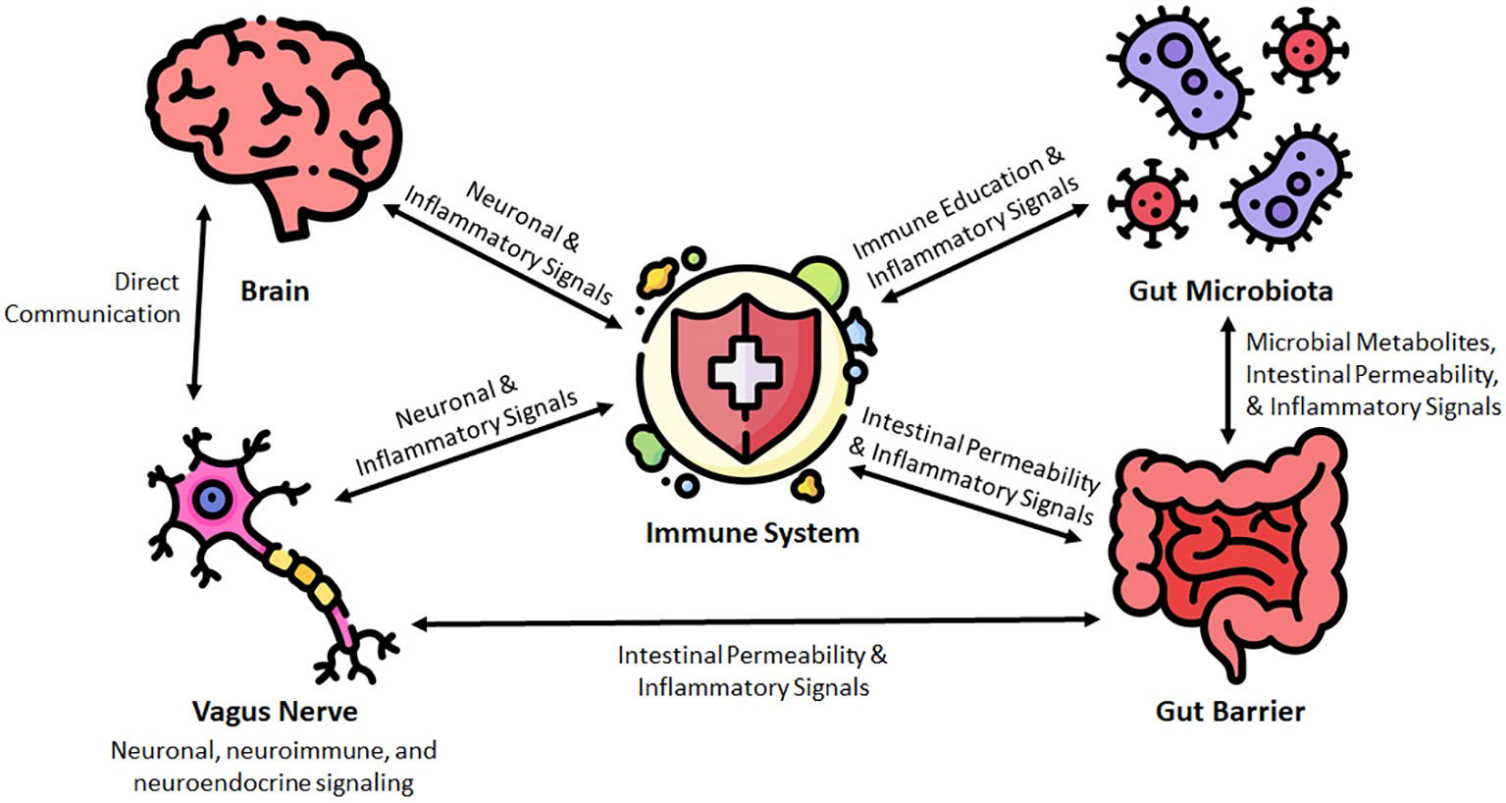
Conceptual framework for the microbiota-gut-brain-immune interface in the pathogenesis of neuroinflammatory diseases.

In the following sections, we discuss the major elements of the microbiome-gut-brain-immune interface. An overview of this relationship can be seen in **Figure 1**.

### 2.1 Microbiome – gut – brain communication

#### 2.1.1 The vagus nerve

Also known as the tenth cranial nerve or ‘the great wanderer,’ the vagus nerve spans the vast majority of the human body and is the key nerve for interoception (internal sensing), and communication between the body and brain to maintain homeostasis or react accordingly (28–30). This communication is bidirectional and involves neuronal, neuroimmune, and neuroendocrine signaling and reaches beyond the parenchyma to include muscle, mucosa, ENS neurons, and the gut microbiome (the gut microbiota and their metabolites, typically measured with metagenomics and metabolomics) (5, 18, 19, 30–32).

#### 2.1.2 Additional vagal connections

The vagus nerve innervates the gut mucosa including the gut associated lymphoid tissue (GALT). Included in the GALT, the lamina propria are part of the immune system and play a crucial role in immune education, which is why numerous immune cells reside here (20). The proximity of the lamina propria to the vagus nerve, (nascent) immune cells, and the gut microbiome make it central to the microbiota-gut-brain-immune interface. Here, the gut microbiota can influence vagal signaling and/or the immune system and therefore affect brain health (30). For instance, bacterial taxa (i.e. *Campylobacter jejuni, Lacticaseibacillus rhamnosus JB-1* a.k.a. *Lactobacillus rhamnosus/reuteri JB-1, Limosilactobacillus reuteri* a.k.a. *Lactobacillus reuteri*) have been shown to affect brain, cognition, and behavior via vagal signaling, resulting in positive and negative outcomes (28, 33). Another demonstration of the interplay between the vagus nerve, the brain and the gut microbiota is found in preclinical data obtained from rodent models. Performing subdiaphragmatic vagotomy on rodents treated with cuprizone blocked demylination in the brain and restored the gut microbiota dysbiosis induced by cuprizone (34). This supports the concept that the vagus nerve plays a critical role in the microbiota-gut-brain axis.

Further, the vagus nerve regulates peripheral inflammation and intestinal permeability via the ENS cholinergic anti-inflammatory pathways, and, therefore, plays a key role in the prevention or pathogenesis of so-called ‘leaky gut’ (31). In times of stress or disease, vagal signaling is inhibited (low vagal tone), which hampers the microbiota-gut-brain-immune interface and can results in negative outcomes in the microbiome, gut, brain, and immune system (31). Extra-vagal signaling in the microbiota-gut-brain-immune interface involves microbial metabolites like SCFAs, secondary bile acids, and tryptophan metabolites including the neurotransmitter serotonin a.k.a. 5-hydroxytryptamine (5-HT) and the gut hormones cholecystokinin (CCK), glucagon-like peptide-1 (GLP-1), and peptide YY (PYY), which are propagated via enteroendocrine cells (EECs) (31, 35). However, stress and disease also affect the gut microbiota and their metabolites; therefore, altering extra-vagal signaling as well.

#### 2.1.3 Secondary bile acids

Bile acids are synthesized from cholesterol. Primary bile acids like chenodeoxycholic acid (CDCA) and cholic acid (CA) are produced first (36). After production in the liver, primary bile acids are transported by way of the small intestine to the colon where they are metabolized by the gut microbiota into the secondary bile acids such as lithocholic acid (LCA) and deoxycholic acid (DCA) (36). These secondary bile acids are then transported across the gut barrier where they may travel to the liver or circulate systemically, likely also crossing the blood-brain barrier (36). While the gut microbiota determines the production of secondary bile acids, secondary bile acids also seem to alter the composition of the gut microbiome, indicating a bidirectional relationship (35, 37). In the brain, no less than 20 bile acids have been found, where they likely alter neurological function and promote disease (36, 37). In a healthy, normal state, bile acids appear to be neuroprotective in the brain; however, in a dysbiotic and/or diseased state, this metabolism and regulation is perturbed, degrading or eliminating the neuroprotective effect (36, 37). Bile acids interact with receptors like the farnesoid X receptor (FXR) and G-protein-coupled bile acid receptor 1 (GPBAR1), which are present in many different immune cells, thereby influencing neuroinflammation (38). Secondary bile acids can also exert an indirect influence on neurological function; for example, LCA and DCA can modulate serotonin production by interacting with the enterochromaffin cells (ECCs) of the gut, thus impacting gut-brain axis signaling (39). The dysregulation of secondary bile acids has been correlated with neurodegenerative disorders such as Alzheimer’s disease, Parkinson’s disease, Amyotrophic lateral sclerosis, and Multiple Sclerosis (40, 41) Secondary bile acids are one of many elements of the microbiota-gut-brain-immune interface that appears to play a major role in the pathogenesis of neurodegenerative disease.

#### 2.1.4 Short-chain fatty acids

Many of the bacterial members of the gut microbiome rely on microbiota-accessible carbohydrates (MACs)—fiber and resistant starch—for fuel, producing metabolites in the process. Of these metabolites the most well-known and, perhaps, beneficial class is the SCFAs, which are an energy source for colonocytes and, therefore, support gut barrier function, microbiome balance, and reduce neuroinflammation, likely to within ‘optimal’ ranges (23, 42). Further, SCFAs promote tolerance and homeostasis via regulatory T cells (Tregs) among many other effects on the immune system and are, thus, considered anti-inflammatory (43–45). Communication between the gut microbiota-gut-brain axis is also mediated by SCFAs via receptors on ECCs, a type of EEC that 1) is involved in serotonin production and 2) directly interacts with the vagus nerve (20, 35).

As with most elements of biochemistry and nutrition, there is an optimal range of SCFAs, both above and below which negative health outcomes are seen. In dysbiotic and/or diseased states, SCFAs are produced in too much (e.g. irritable bowel syndrome) or too little (e.g. low MACs diet) quantities. Some of the SCFAs are transported across the gut barrier and into circulation, where they appear to cross the blood-brain barrier, affecting CNS function (46). SCFAs such as butyrate have been shown to promote gene expression via epigenetic regulation (i.e. enhanced chromatin accessibility), which may aid in memory consolidation (46). However, not all SCFAs are created equal. For example, butyrate is particularly neuroprotective via Treg induction while acetate may exacerbate neurodegeneration (23).

#### 2.1.5 Neurotransmitters

Transmitting electrochemical signals between neurons and to effector sites, neurotransmitters can act as hormones, promoting function and health in peripheral tissues including the brain (47, 48). Many neurotransmitters are also produced by gut microbiota including γ-aminobutyric acid (GABA), norepinephrine, epinephrine, dopamine, and acetylcholine (20, 49, 50). While these microbial neurotransmitters clearly play a role in the gut (see **Tables 1**, **2**), in proximal regions, and likely in circulation, it is unclear if all or some of these interact with the CNS in sufficient concentrations to have a meaningful effect; however, they can exert an effect via the ENS including the vagus nerve (51, 52). Further, SCFAs may add to this effect on vagal signaling, as SCFAs play a key role in neurotransmitter metabolism; for example, SCFAs modulate the production of tryptophan by ECCs. Tryptophan is a required pre-cursor to serotonin (20).

**Table 1:** Bacterial effects on neurotransmitters, by neurotransmitter.

Neurotransmitter	Observation	Bacterial Taxa*
Acetylcholine	Support/Produce	*Lactobacillus*
Acetylcholine	Support/Produce	*Bifidobacterium*
Acetylcholine	Support/Produce	*Enterococcus*
Acetylcholine	Support/Produce	*Streptococcus*
GABA	Support/Produce	*Lactobacillus*
GABA	Support/Produce	*Bifidobacterium*
GABA	Support/Produce	*Enterococcus*
GABA	Support/Produce	*Streptococcus*
GABA/glutamate	Metabolize intermediate	*Lactobacillus* spp.
GABA/glutamate	Metabolize intermediate	*Bifidobacterium* spp.
Serotonin	Produce	*Lactobacillus*
Serotonin	Produce	*Bifidobacterium*
Serotonin	Produce	*Streptococcus*
Serotonin	Downregulate	*Enterococcus*

*This is likely not the case for all species/strains but has been observed within this domain.

**Table 2:** Bacterial effects on neurotransmitters, by taxa.

Bacterial Taxa*	Observation	Neurotransmitter
*Bifidobacterium*	Support/Produce	Acetylcholine
*Bifidobacterium*	Support/Produce	GABA
*Bifidobacterium*	Produce	Serotonin
*Bifidobacterium* spp.	Metabolize intermediate	GABA/glutamate
*Enterococcus*	Support/Produce	Acetylcholine
*Enterococcus*	Support/Produce	GABA
*Enterococcus*	Downregulate	Serotonin
*Lactobacillus*	Support/Produce	Acetylcholine
*Lactobacillus*	Support/Produce	GABA
*Lactobacillus*	Produce	Serotonin
*Lactobacillus* spp.	Metabolize intermediate	GABA/glutamate
*Streptococcus*	Support/Produce	Acetylcholine
*Streptococcus*	Support/Produce	GABA
*Streptococcus*	Produce	Serotonin

*This is likely not the case for all species/strains but has been observed within this domain.

Serotonin has been extensively studied for its role in gastrointestinal and brain health and in gut-brain communication (the gut-brain axis) (51). The majority of serotonin (~90%) is stored in ECCs in the gut, produced from tryptophan (47, 53, 54). Tryptophan to serotonin metabolism involves the kynurenine pathway and, therefore, leads to production of quinolic acid (neurotoxic) and kynurenic acid (neuroprotective)—the balance of which is regulated by the gut microbiota and may contribute to neuroinflammation and ultimately neurodegeneration (55). While neurotransmitter-producing gut microbiota are still in the early stages of elucidation, there are a few bacterial taxa of note (see **Tables 1**, **2**).

Throughout the body, the microbiota-gut-brain axis and subsequently the microbiota-gut-brain-immune interface has a profound impact via direct and indirect pathways and is influenced by host genetics, lifestyle, environmental exposures, etc. As many of these are modifiable risk factors, this is an important line of research to support mental and physical health (well-being) and may prove to be crucial for prevention, management, and treatment of neuroinflammatory and neurodegenerative disorders, for which we currently have very few tools at our disposal.

## 3 Neuroinflammation

Physical and/or psychological stress can also cause inflammation, including chronic neuroinflammation, through dysregulation of the hypothalamic-pituitary-adrenal axis (HPA axis). Inflammation can originate anywhere in the body, typically via inflammatory cascades that include cytokines, chemokines, reactive oxygen species (ROS), and trafficking of immune cells (e.g. T and B cells). Neuroinflammation also involves specialized members of the immune system called glia (microglia and astrocytes) resident in the CNS. A robust, acute immune response is a necessary response to injury or invasion; it is equally important for this inflammation to resolve in a timely manner, avoiding chronic inflammation. This is true of neuroinflammation as well. While acute neuroinflammation is protective, chronic neuroinflammation increases risk of neurodegenerative disorders, the most common of which are Alzheimer’s disease, Parkinson’s disease, prion disease, amyotrophic lateral sclerosis, motor neuron disease, Huntington’s disease, spinal muscular atrophy, and spinocerebellar ataxia (56). Further, local or systemic inflammation can sensitize the immune system, leading to exacerbation of inflammation, including neuroinflammation.

Gut microbiota influence brain function by way of maintenance of homeostasis in innate and adaptive immunity, limiting acute and chronic inflammation in the gut and CNS and, therefore, risk of neurodegenerative disorders even independent of other pathogenesis features like amyloid plaques (23, 55, 57). Neuroinflammation is a key element in the pathogenesis, prevention, and treatment of neurodegenerative disorders; once at a critical point, the epigenetic profile changes dramatically (13). Epigenetic changes due to neuroinflammation may include substantial changes in DNA methylation, histone methylation and acetylation, and non-coding RNA expression (13). Further, the relationship between neuroinflammation and epigenetic changes has long suspected to be bidirectional, as neuroinflammation is also strongly influenced by epigenetic mechanisms. For instance, DNA methylation may be a regulator of activated microglia that drive AD pathology, and presence of neuroinflammatory conditions (e.g. psychiatric disorders) demonstrate altered patterns of DNA methylation (58). The origin of neuroinflammation is often outside of the CNS, commonly certain bacterial taxa in the gut microbiome. For example, *Heliobacter pylori*, which is present in about half of human gut microbiomes, leads to DNA methyltransferase inhibition, destabilizing the genome in a manner typical of certain disease states (46).

It is important to note the possibility that dysbiosis resulting in altered protein express may also contribute to neuropathology, independent of the inflammatory response. In a mouse model, fecal microbiota transplant (FMT) from aged donors to young adult mice resulted in impaired spatial memory in conjunction with altered protein expression associated with hippocampal synaptic plasticity and neurotransmission with concomitant reduction in SCFA-producing bacteria; yet, gut permeability and cytokines were not affected (59). However, the authors note that cytokines were assessed at the end of the intervention and were unaware if cytokines fluctuated in the early-stage post-FMT.

The role of vascular endothelial growth factor (VEGF) may also serve an underrecognized role in the multifactorial pathophysiology related to alteration of the gut microbiome, protein expression, and inflammation. Of note, in AD mouse models in which alterations in RNA/protein expression and microglia occur with elevated amyloid-beta-peptides in the ENS of the small intestine, some evidence suggests that VEGF mediates neuroprotective and neurodegenerative effects in both the CNS and PNS (60).

### 3.1 Glia: astrocytes and microglia

These immune cells reside in the nervous system; in the CNS glia are involved in the production, potentiation, and resolution of neuroinflammation. Also known as glial cells or neuroglia, glia support neuronal functions, e.g. synapse formation, neuronal plasticity, neurotransmission, injury response, and protection from neurodegenerative disorders. Glia release cytokines and chemokines that are potential mediators of neurotoxicity (**Table 3**). The most plentiful glia are astrocytes, which are “master regulators of synapse formation, ion homeostasis, and neurovascular coupling” (13).

**Table 3:** Immune response via glia.

	Relevant Roles
Cytokines (secreted by immune cells)	
Interleukins (IL)	
•IL-1β	Crucial for host defense against pathogens but can also worsen damage in chronic disease and acute tissue injury. It aids in combating microbes and facilitating tissue repair mechanisms.
•IL-6	Regulates innate immunity and initiates inflammation. Known to contribute to pain, hypersensitivity, neuropathy, and cancer by interacting with immune cells, glia cells, and neurons along the pain pathway.
•IL-8	A chemoattractant cytokine. Produced by various tissue and blood cells. Uniquely targets neutrophils (minimal effects on other blood cells) specifically in inflamed areas.
•IL-33	Produced by synapse-associated astrocytes; essential for normal synapse numbers and circuit function in the thalamus and spinal cord. Primarily signals via microglia to enhance synaptic engulfment under normal conditions. In mice, hippocampal IL-33 triggers inflammation, resulting in cognitive impairment.
Tumor necrosis factor-alpha (TNF-α)	3 interconnected vicious cycles: 1) Microglia release TNF-α, which stimulates release of TNF-α and glutamate, activating microglial receptors, leading to more TNF-α release. 2) TNF-α prompts astrocytes to release glutamate, which accumulates due to inefficient uptake, raising extracellular glutamate. 3) TNF-α disrupts the balance of synaptic activity, causing excessive calcium entry and neuronal death; dying neurons sustain microglial activity, further increasing TNF-α release.
Chemokines (cytokines that attract immune cells)	
CCL2 a.k.a. monocyte chemoattractant protein-1 (MCP-1)	Pro-inflammatory mediators that attracts or enhances the expression of other inflammatory factors/cells. Up-regulated in many central nervous system (CNS) disorders with blood brain barrier (BBB) breakdown.
CCL5 a.k.a. Regulated upon Activation, Normal T cell Expressed and presumably Secreted (RANTES)	

In response to changes in their environment, in addition to producing cytokines and chemokines, microglia express antigenic markers, regulate neurotransmitters, and undergo morphological changes (61). Once activated, microglia can cause neuronal damage by producing reactive oxygen species and nitric oxide—both neurotoxic—and cross-reacting with astrocytes to magnify the effect, resulting in loss of neurotrophic functions. Microglia have been proposed to promote neuroinflammation and neurotoxicity; however, recent research suggests that their impact can be context-dependent, contingent upon their polarization phenotype, activation status, and cellular context (62). These effects are modulated through neuron-microglia communication facilitated by various neurotransmitter receptors expressed on microglia. Notably, receptors for neurotransmitters such as glutamate, GABA, norepinephrine, cannabinoid, and acetylcholine play significant roles in mediating these interactions. Microglia may modulate neurotransmitter release, thus, coordinating either positive or negative feedback loops tailored to the needs of the organism. Moreover, these interactions may extend to indirect effects on neighboring microglia, further expressing the role of neuron-microglia communication.

### 3.2 Chronic neuroinflammation

The relationship between the microbiota-gut-brain-immune interface, microbial metabolites, and glia is a potent regulator of GI and actionable target in neuroinflammatory and neurodegenerative disorders (12). In chronic neuroinflammation, pro-inflammatory cytokines are habitually upregulated and glia are overactive, resulting in damage to neurons, synapse function, cortical tissue, and functional connectivity—commonly observed in neurological disorders (57, 63). The multidirectional relationship of the microbiota-gut-brain-immune interface governs this chronic neuroinflammation, which may start at any point, including moving from the gut into the CNS, which often results in systemic inflammation. Systemic inflammation is a core feature of chronic neuroinflammation. Neuroinflammatory diseases arecharacterized by elevated C-reactive protein (CRP), TNF-α, and IL-1β (23). The overactivation of glia that drives pathogenesis is affected by the gut microbiome and implicated in neuroinflammation and subsequent neurological disorders (e.g. Alzheimer’s and Parkinson’s), as well as gut microbiome composition (dysbiosis) and intestinal inflammation and permeability (‘leaky gut’) (18, 23, 64). Neurodegenerative disorders are likely multifactorial in cause, and a key element of this is the gut microbiota due to their production of neuroactive metabolites (5). In specific, gut microbiota that produce the endotoxin lipopolysaccharides (LPS) may contribute to amyloid deposition and neuroinflammation in Alzheimer’s (65). LPS interacts with the microbiota-gut-brain-immune interface via Toll-like receptor (TLR) 4 and the NF-κB pathway, stimulating an inflammatory cascade, triggering leaky gut, and leading to neuroinflammation (18, 23, 64–66).

Interleukins (ILs) are a category of cytokines: some of which have pro-inflammatory/immune reaction stimulating effects while others have anti-inflammatory/homestasis stimulating effects. Therefore, the change in the concentrations of these cytokines (the cytokine milleau) can have great effects on neuroinflammation. ILs are a promising target for treating neuroinflammation and subsequent neurodegeneration (67). Many of the Preventative and Therapeutic Interventions discussed below alter the cytokine milleau, meaning this is at least in part their method of action.

TLRs are pattern recognition receptors (PRRs) that recognize molecules and patterns of molecular structure, pathogen-associated molecular patterns (PAMPs), from bacteria that are extracellular or have been engulfed into vesicular pathways via phagocytosis. They signal through cytokines such as ILs. TLRs could be used as targets to quench neuroinflammation, according to research from preclinical to clinical trials (68). Futher, small phytocompounds such as curcumin have been shown to target TLRs (68). The dose and other details for such an intervention have yet to be elucidated and show the possiblity of a hormetic response, meaning higher doses are actually detrimental.

No matter the cause(s), dysbiosis fosters a damaging, inflammatory environment via the microbiota-gut-brain-immune interface via LPS and other stimulators of the inflammatory cascade, i.e. pro-inflammatory cytokines and chemokines, T-helper cells, and monocytes (5). Further, dysbiosis contributes to aberrant HPA axis activation that can result in cortisol dysregulation, which exacerbates leaky gut (5). Therefore, dysregulation of the microbiota-gut-brain-immune interface can create a circular inflammatory feedback loop between dysbiosis, leaky gut, and chronic systemic and CNS inflammation.

## 4 Microbial alterations in neuropathology

The emerging patterns of gut microbiome changes specific to neurological disorders may aid in the development of treatment options for these recalcitrant disorders (described in subsequent sections). This data is largely correlational and focused solely on the composition of the gut microbiome, meaning it is not yet ready for the clinic. Further, some of this compositional work has been done at fairly high order such as phyla/phylum, which is extremely non-specific. While other work is done at the genus level, this is still fairly non-specific with a great deal of diversity of function within a single genus. Keeping this in mind, one must take the research on the composition of the microbiome, especially that at the phyla or genus level, with more than a grain of salt. Functional data on the gut microbiome are beginning to emerge and will grow substantially in the years to come as the cost of advanced technologies such as shotgun metagenomics continue to decline and are adopted more widely. This coupled with more robust study designs including longitudinal studies may lead to groundbreaking therapies and even means of prevention.

Currently, it appears that there are common mechanisms among neurodegenerative disorders like Alzheimer’s disease, Parkinson’s disease, multiple sclerosis, and stroke/traumatic brain injury. These include a dysbiotic gut microbiome, insufficient SCFA production, elevated LPS, and leaky gut that stimulate pro-inflammatory immune, neuroendocrine, and neuroinflammatory pathways. It is likely that the differences between these disorders is due to variation in the microbial alterations of the affected gut microbiomes. This may or may not also be related to baseline microbiome composition and function prior to the onset of neuroinflammation, which are largely determined by environmental exposures including diet and lifestyle in addition to seeding of the gut microbiome during crucial developmental phases as well as host genetics (e.g. propensity for chronic inflammation) (25, 69). These relationships are summarized in **Supplementary Table S1**.

### 4.1 Alzheimer’s disease

The hallmarks of Alzheimer’s disease are amyloid-beta and hyperphosphorylated tau protein accumulation with neuronal degeneration, which is thought to develop decades prior to symptoms. The microbiota-gut-brain-immune interface has been implicated in its pathogenesis with human studies finding dysbiosis in the gut microbiomes of those with Alzheimer’s (69–71). For example, the gut microbiomes of those with Alzheimer’s compared to healthy controls has decreased Bacillota (formerly Firmicutes) and *Bifidobacterium* and increased Bacteroidota (formerly Bacteroidetes), and Escherichia and Shigella (two inflammatory genus) as well as lower abundance of the species *Eubacterium rectale* (*E. rectale* is believed to be anti-inflammatory), all of which correlates with increased pro-inflammatory cytokines in Alzheimer’s (72, 73). However, these compositional changes have not been confirmed in cohorts in other countries, such as China, indicating a role for the environment and/or the need for higher resolution data, likely at the species or strain level (74, 75). There is a need for further research, especially that better control for potential confounders, to be able to use this as a screening tool for research or clinically.

### 4.2 Parkinson’s disease

In Parkinson’s disease, misfolded α-synuclein accumulates in the neuronal cell body with motor impairments at least in part due to progressive dopaminergic neuron damage, resulting in decreased dopamine (5, 76). Of note, digestive symptoms are common in Parkinson’s and typically develop prior to hallmarks of the disease. This points to a key role of the microbiota-gut-brain-immune interface and lends some support for a causative role via temporality *à la* the Bradford Hill criteria (5, 21, 77). While it is not yet clear that α-synuclein causes neuronal loss (it may be an intermediate step or a symptom), the vagus nerve may transmit pathology to the brainstem, resulting in deposition of α-synuclein (78, 79). In fact, correlations have been established between α-synuclein and composition of the gut microbiome, which have been supported by a recent meta-analysis (76). Specifically, compared to healthy controls, the gut microbiomes of those with Parkinson’s have: depletion of *Prevotellaceace* that are involved in SCFA production, which leads to leaky gut and endotoxin exposure; depletion of important SCFA producers belonging to the Lachnospiraceae family and of which key players include *Blautia*, *Roseburia*, and *L-Ruminococcus*; increased *Enterobacteriaceae*, which can raise LPS and eventually lead to neuroinflammation; and enrichment of *Lactobacillus*, *Akkermansia*, and *Bifidobacterium* (5, 76). However, it is unclear if these changes are the cause or consequence of disease at present.

### 4.3 Multiple sclerosis

Gut dysbiosis appears to promote the pathogenesis of Multiple Sclerosis (MS) via the microbiota-gut-brain-immune interface: leaky gut, leading to immune activation, leading to systemic inflammation, leading to disruption of the blood brain barrier, leading to neuroinflammation, leading to neurodegeneration (52, 80). The impetus for this cascade and the leaky gut that drives it is currently unknow but dysbiosis has been found in MS patients and is mechanistically plausible (52, 80–83). While it is possible that this may actually be a consequence of the disease process rather than its cause, the research to date does not clearly support this concept. Further, the types of MS seem to have their own distinct versions of dysbiosis (52, 80–83). When compared to healthy controls, the gut microbiomes of those with MS have fewer SCFA-producing bacteria, *Butyricimonas*, *Faecalibacterium*, *Clostridium* cluster IV and XIVa, *Faecalibacterium prausnitzii*, and Blautia species and more *Akkermansia muciniphila*, *Ruthenibacterium lactatiformans*, *Hungatella hathewayi*, *Eisenbergiella tayi*, and *Clostridium perfringens* (82–84).

### 4.4 Overlap in neurodegenerative disorders

In a 2022 systematic review involving 52 studies and 5,496 participants with Alzheimer’s disease, Parkinson’s disease, MS, amyotrophic lateral sclerosis, and stroke, the strongest overlap was seen between Parkinson’s disease and MS with 8 shared genera (85). Interestingly, Parkinson’s also shared 6 genera with stroke. While there was overlap between Alzheimer’s and Parkinson’s the sample size is small, making this an unreliable association at present. Among these CNS disorders, *Akkermansia*, *Faecalibacterium*, and *Prevotella* were most commonly indicated. Again, this work is done at high order (low resolution) and is still mostly correlational and, therefore, may not be causative. However, these overlapping trends may better inform researchers and clinicians about preventative and interventional measures that could be more broadly applicable to neuroinflammatory disorders. Thus, this may represent a research priority for funding agencies. Key relationships for all of these disorders are highlighted in **Supplementary Table S1** *Observed Functional Relationships with the Gut Microbiota and Neurodegenerative Disorders*; of note, only bacterial taxa have been characterized sufficiently at present.

### 4.5 Preventative and therapeutic interventions

Much of the excitement around the research on the microbiota-gut-brain-immune interface, especially around altering the gut microbiome, is the possibility of preventative and therapeutic interventions. This is especially poignant in neurodegenerative disorders, where there is little to offer in terms of such interventions. Most of the research has been in animal models with limited human studies, however. Translating these findings into the clinic requires further investigation in general and in how best to personalize such interventions to maximize their impact for an individual.

### 4.6 Nutrition for neuroinflammation

Nutrition is an important, modifiable risk factor that has a major role in the microbiota-gut-brain-immune interface, affecting each aspect of the interface. Gene-diet interactions have been linked to the microbial theory of inflammation, neuroinflammation, and neurodegenerative disorders like dementia (86, 87). Diet induced changes to the gut microbiome are key in the microbiota-gut-brain-immune interface. Gut microbiome changes are associated with shifts in the production of SCFAs and secondary bile acids, which in turn can impact inflammation and the release of neurotransmitters like serotonin (35). Therefore, resolving inflammation, dysbiosis, and leaky gut are likely to prevent and/or manage neurodegenerative disorders. The quantity of MACs present in the diet is linked to the production of SCFAs (88). A diet with adequate calories, rich in MACs promotes health in the microbiota-gut-brain-immune interface, while a high calorie, low MACs diet is associated with cognitive decline (25, 89, 90). SCFAs clearly play a role in this, especially in light of their role as fuel for colonocytes, preventing leaky gut and the inflammatory cascade (25, 89, 90). Polyphenols may also play a key role and have been shown continually and repeatably to be health-promoting elements of a healthy diet (25, 91, 92). Thus, diets low in MACs, high in fat/protein (i.e. Western-style diets) are associated with gut dysbiosis and inflammation (25, 93). While some research on ketogenic diets, which are often low in MACs, has shown promise in reducing neuroinflammation and improving cognitive function in animal models of Alzheimer’s and Parkinson’s diseases, microbiome research poorly translates from animal models, which are overly simplistic. Therefore, the results in humans are often greatly attenuated or even lost due to a much more complex physiology, meaning they are no longer meaningful let alone clinically meaningful. This necessitates research in humans, which is currently lacking for ketogenic diets.

The nutrients and nutraceuticals often recommended for brain health mostly support the gut microbiome and the barrier function of the gut and blood brain barrier (94). However, any individual food is more than the sum of its parts—the concept of the ‘food matrix’ (95–98). Therefore, focusing on nutrients alone is insufficient to promote a healthy diet. Instead, an emphasis on the inclusion of whole, minimally processed foods and limiting ultra-processed foods is necessary and likely better able to support the microbiota-gut-brain-immune interface and prevent the inflammatory cascade and consequently neurodegeneration. Dietary patterns that embody this include the Mediterranean diet and the Mediterranean-DASH Diet for Neurodegenerative Delay (MIND) diet.

The Mediterranean diet is a style of dietary pattern that emphasizes vegetables, fruit, olive oil, and low-moderate alcohol intake (i.e. red wine) and is considered to be health promoting. It is also rich in polyphenols and omega-3 fatty acids, the latter of which is required for the resolution of the inflammatory response via resolvins and are associated with reduced neuroinflammation (99, 100). This dietary pattern has been linked to lower risk of neurodegenerative disorders and cognitive impairment and better global cognition and episodic memory (101). Many of the disorders that are seen to be decreased in those on this type of diet (coronary artery disease, hypertension, diabetes, metabolic syndrome, dyslipidemia) are also risk factors for cognitive impairment and involve the microbiota-gut-brain-immune interface (99).

The MIND diet is a version of the Mediterranean diet with an emphasis on neuroprotection and cardioprotection through anti-inflammatory foods that has been shown to slow cognitive decline with aging (99, 101, 102). It consists of high intake of whole plant foods emphasizing berries and green leafy vegetables, nuts, beans, fish, poultry, and olive oil while limiting animal foods, processed foods, and foods high in saturated fat. The focus on the food matrix likely plays a role in the MIND diet’s effect on the microbiota-gut-brain-immune interface, potentially making it a more comprehensive tool.

Adherence to both the Mediterranean and MIND diets are associated with decreases in all-cause dementia independent of genetic risk and numerous studies support reduction in the risk of Alzheimer’s disease specifically (19, 86, 103). Both the Mediterranean and MIND diets are associated with reduced pathology in Alzheimer’s (104). Evidence to date demonstrates support for the Mediterranean and MIND diets in the prevention of a multitude of disease states, and the MIND diet appears to impart the greatest neuroprotection. However, there are limitations to this research: small cohorts, lacking a gold-standard to measure dietary adherence, potential for reverse causality because of short durations/follow-up (86, 105). In an attempt to account for this, a recent population-based study by de Crom et al. found an association between both diets and reduced dementia risk; however, there is still potential for confounding from lifestyle (105).

#### 4.6.1 Lifestyle modifications

Many modifiable risk factors fall under the category of lifestyle. Stress management, restorative sleep, and other lifestyle factors have been shown to affect epigenetic regulation, the microbiota-gut-brain-immune interface, and neurodegenerative disease risk (106–112). As discussed above, chronic stress can trigger gut dysbiosis and the inflammatory cascade, so it follows that stress management has been linked to improvements in the gut microbiome composition and in stress-related epigenetic regulation (93, 113, 114). Mind-body therapies, e.g. yoga and meditation, are multifaceted interventions with numerous health benefits including stress management. Such mind-body interventions are promising to promote a diverse, non-dysbiotic/eubiotic gut microbiome and to reduce chronic inflammation (115–118). Similarly, restorative sleep is negatively associated with gut dysbiosis and cognitive decline (119, 120).

#### 4.6.2 Physical activity

Effecting both the gut microbiome and epigenetics, physical activity is a potentially powerful modifier of neurodegenerative disorder risk. Physical activity (natural movement and exercise) alters gut composition and function by promoting beneficial gut bacteria and SCFA production; it also supports resolution of inflammation and return to immune homeostasis (121–125). All of these effects are likely to reduce the risk of neurodegenerative disorders. Additionally, physical activity is generally neuroprotective: increasing cerebral blood flow and circulation-related benefits (i.e. oxygenation and nutrient delivery), the production of neurotrophic factors including brain-derived neurotrophic factor (BDNF), and neurotransmitter (e.g. dopamine and serotonin) release that improves mood, cognition, and well-being (126–128). BDNF promotes neuronal growth, survival, and synaptic plasticity, supporting learning and memory (127). This orchestrated interplay of neurotrophic, anti-inflammatory, and metabolic processes work hand in hand with the gut microbiome in conferring neuroprotection associated with physical activity. Epigenetic changes from physical activity also contribute to its neuroprotective role (106, 112, 128–130). Given that yoga is both exercise and a mind-body therapy, both processes are likely contributing to the beneficial effects of yoga on the microbiota-gut-brain-immune interface and therefore reduced risk of neurodegenerative disease.

#### 4.6.3 Probiotics, prebiotics, and fermented foods

Probiotics are, by definition, live microorganisms that confer health benefits when administered in adequate amounts (131). In animal models of neurodegenerative disorders, supplementation with probiotics shows great promise for improving neuroinflammation, cognitive function, gut microbiome composition and function, epigenetic profiles, inflammation, and gut barrier function (132–139). A better understanding of the dysbiosis in neurodegenerative diseases as well as how certain taxa (e.g. keystone species) affect the microbiota-gut-brain-immune interface is necessary to rationally design probiotics that may be preventative or therapeutic for neurodegenerative disorders. At present, the majority of probiotic strains on the market are taken from yogurt, as they are easily granted generally recognized as safe (GRAS) status by the Food and Drug Administration (FDA). This greatly limits the possibility of their efficacy as therapeutics in an ecosystem that is much more diverse than yogurt.

Prebiotics are MACs that stimulate the growth and activity of microbes already present in the gut microbiome, thereby also altering the composition and function of the gut microbiome (140). Prebiotics have been shown to increase beneficial gut bacteria, reduce neuroinflammation, and support cognitive function (134, 137, 139). Given that prebiotics are likely to have a broader effect than the limited types of probiotics currently available, this may be a more promising avenue. However, a healthy diet emphasizing whole foods and limiting ultra-processed foods (e.g. the Mediterranean or MIND diets) can supply sufficient MACs to fuel the gut microbiota. Hence, the use of prebiotics over the emphasis of a healthy diet, which brings many other health-promoting elements, is currently under debate. In those unwilling or unable to adopt a diet rich in MACs, it is possible that prebiotics may support the gut microbiota sufficiently to avoid neurodegenerative disorders or it may be insufficient or missing other key elements from the diet and the food matrix.

The microbiota-gut-brain axis involves bidirectional communication through multiple pathways. These pathways, both direct and indirect, can facilitate epigenetic reprogramming within the microbiota-gut-brain axis, mediated by histone tail modification, DNA methylation, and non-coding RNA. Alterations in the composition of the microbiota can induce epigenetic changes that ultimately influence behavior; for example, *Helicobacter pylori* in the gut increases CpG-methylation in the promoter region of 06-methylguanine DNA methyltransferase, consequently reducing DNA methyltransferase activity in the gastric mucosa (141). Furthermore, studies have discovered a correlation between the gut microbiome and gene expression within the CNS, particularly in regions controlling the development of mood and neurological disorders; for instance, dietary supplementation of mice with *Lactobacillus rhamnosus* has been found to modulate the expression and transcription of GABA subunits across various brain regions (141). Interventions involving the supplementation of pre- or probiotics may ameliorate neurobehavioral abnormalities through epigenome alteration, often resulting in phenotypic attenuation.

Fermented foods were traditionally used to extend the storage of perishable food substrates; however, recent studies have highlighted their roles in the introduction of beneficial microbes and molecules to the gut microbiome. The connecting pathways of the microbiota-gut-brain axis have been used to understand the effects of fermented foods on the permeability of the intestinal and blood-brain barrier and their role in the treatment of neuroinflammation and mental health disorders. Various studies have demonstrated a decrease in circulating cytokines, especially IL-6, IL-10, IL-12, and TNF-α, among patients on fermented food diets (142). Consumption of fermented foods has also been shown to reduce corticosterone when exposed to stress; a proposed mechanism of cortisol modulation is via attenuating the response to peripheral immune challenges through a reduction in circulating cytokines and other inflammatory mediators (142). The administration of fermented products has also been shown to improve anxiety and depressive features and improved memory-associated tasks (142).

#### 4.6.4 Other supplements

##### 4.6.4.1 Gut barrier: glutamine and zinc carnosine

Supplements that support gut barrier function include glutamine (143–145) and zinc carnosine a.k.a. polaprezinc, which is important for wound and mucosa (e.g. the gut) healing and likely also beneficial for neuroprotection and reducing neurodegenerative disorder risk (146–149).

##### 4.6.4.2 inflammation

###### 4.6.4.2.1 Omega-3 fatty acids

As mentioned previously, omega-3 fatty acids help to resolve inflammation (anti-inflammatory); thus, supplemental omega-3 may be beneficial for the microbiota-gut-brain-immune interface. In fact, a reduced risk of Alzheimer’s disease and cognitive decline is associated with intake of omega-3, especially docosahexaenoic acid (DHA) (150). However, this effect has not been consistent perhaps due to issues with dose, formulation, rancidity, study design, etc. (151, 152). In Parkinson’s disease, several studies have demonstrated a reduction in dopaminergic neuron degeneration and neuroinflammation with greater intake of omega-3 (151).

###### 4.6.4.2.2 Curcumin

A polyphenol found in turmeric, curcumin’s anti-inflammatory properties have been studied as a potential therapeutic in Alzheimer’s disease with the exploration of several modified formulations include nanotization to improve its bioavailability and pharmacokinetic properties, the major limitation to its therapeutic benefits (153–155). However, given the food matrix/entourage effect, one wonders if the extract is as potent as the whole food (turmeric) and/or if there are synergistic effect in food combinations. For instance, it is well known that black pepper improves the bioavailability and action of turmeric (156–158).

###### 4.6.4.2.3 Resveratrol

Another polyphenol, resveratrol and its sources (i.e. red grapes and wine) have been linked to improved cognitive function and neuroinflammation and are being studies for Alzheimer’s disease and Parkinson’s disease (159–163). However, resveratrol research is still in its early stages and has hit some barriers, including concerns for the need for high doses. Again, the food matrix/entourage effect may be an important component to explore, potentially limiting the need for high dose therapy.

#### 4.6.5 Fecal microbiota transplantation

Fecal Microbiota Transplantation (FMT) is the transfer of fecal matter from a healthy donor into the gut of a recipient after the administration of antibiotics to clear the way (164). While historically thought of as radical and reserved for the life-and-death struggle of recurrent *Clostridioides difficile*, FMTs are being developed by industry, including two that have been FDA-approved recently (164–167). These standardized FMTs open up the possibility of broader use including for neurodegenerative disorders (168–172). To understand the long-term safety and efficacy of FMT in neurodegenerative disorders and for neuroinflammation, more research is needed.

## 5 Generalizability of microbiome research findings

In an emerging field like this, some demographic and ethnic groups are underrepresented, limiting the generalizability of its findings (173). Why is this so important for the microbiota-gut-brain-immune interface and the pathogenesis of neurodegenerative disorders? In a 2015 study, gut microbiome compositions were shown to vary significantly by geographic location, meaning the composition of those in the US, are likely different than those in other countries and the findings from US research may not be translatable in other countries (174). This has since been confirmed by many other studies and researchers. The gut microbiome is similar to a finger print in that there is a huge amount of interindividual variability, meaning averages are often not representative as well. Therefore, small sample sizes, especially from groups with limited diversity, are only able to describe the population in the sample—they are not generalizable (175). Despite this, high-income countries (HICs) lead microbiome research output due to well-established research infrastructure and funding availability. Contrastingly, lower-middle-income countries (LMICs) are underrepresented in microbiome research (175). Access is limited to advanced sequencing technologies, funding, and expertise, all of which encumber research efforts in LMICs (173, 176). Further, the emphasis on infectious disease and more immediate concerns to public health likely redirect consideration and resources from more long-term research projects such as the microbiome (176).

In order to truly understand the gut microbiome and the microbiota-gut-brain-immune interface, we must study diverse populations. This will ensure the equitable advancement of personalized medicine and healthcare generally. Diverse populations with distinct lifestyles and dietary patterns reside in LMICs. Microbiome studies in these regions are likely to elucidate population-specific variations in susceptibility to disease, response to therapies, and interaction with the environment. Likely barriers in some of the most underrepresented groups are highlighted subsequently.

### 5.1 Sub-Saharan Africa

Perhaps the most underrepresented region in gut microbiome research is Sub-Saharan Africa. With its immense genetic, cultural, and environmental diversity, increasing research in this region will greatly advance our understanding of unique microbial interactions and the related implications for human health. Research in this area is limited largely due to barriers such as insufficient research infrastructure, funding scarcity, access to advanced technologies, and ethical issues such as informed consent and sharing samples (176, 177). In addition to addressing these barriers to gut microbiome research, promoting collaborative research in this field within Africa will lead to priceless insights into the diverse human population in this continent as well as a more complete comprehension of the human microbiome globally.

### 5.2 Latin America

Gut microbiome research remains comparatively limited in Latin America as a region. In contrast, some countries have made advances in this field; however, even these are still lagging behind high-income countries. Major barriers include funding scarcity, insufficient research infrastructure, and inadequate expertise pipelines. Collaboration between Latin American and international researchers can help bridge this gap and foster knowledge exchange to enhance gut microbiome research regionally and globally (178).

### 5.3 Southeast Asia

Due to the large population in Southeast Asia, this region represents one of the most significant for global health research, generally, and gut microbiome research, specifically. Despite this, this region continues to lag behind in gut microbiome research. Barriers include a dearth of well-established research institutions (insufficient research infrastructure) and funding scarcity. As in Latin America, collaboration may fill some of this gap; however, regional capacity building will also be required (179). Both are necessary to advance gut microbiome research in this region and lead to a wholistic understanding of the gut microbiome globally.

### 5.4 Middle East

The final key, underrepresented region in gut microbiome region is the Middle East with relatively limited gut microbiome research (180, 181). Barriers in this region include political instability, funding scarcity, and ethical issues including cultural norms and sharing data. Again, international collaboration can overcome some of this with the ultimate need for local capacity building.

### 5.5 Considerations for improving the generalizability of microbiome research

Capacity building is a key step in addressing the underrepresentation of LMICs in gut microbiome research. To build capacity locally, investments will need to be made to provide training and support to local researchers, equipping them with the latest technology, and developing collaborations between HIC and LMIC institutions. An example of this being success is reported by Maduka et al. in 2017, where African researchers were empowered through bioinformatics training and development of the necessary infrastructure (182).

Another element of support is international partnerships and exchange programs, which can cultivate knowledge exchange and resource sharing. It is common to see large-scale collaborations in HICs (e.g. the Human Microbiome Project), which have facilitated data and resource sharing to advance gut microbiome research with comprehensive datasets and groundbreaking discoveries (141, 183). Some of these HIC projects have promoted international collaboration (i.e. Earth Microbiome Project, American Gut Project, MetaHIT Consortium) and could serve as models for bringing LMICs in as well (184–186).

Additionally, community engagement is indispensable to ensuring appropriate, diverse representation in gut microbiome research. To conduct research in these populations, culturally appropriate approaches must be used, which require respecting cultural practices and beliefs (187). Further, such approaches (e.g. culturally sensitive recruitment strategies and community-based participatory research design) promote diversity, equity, and inclusion in the research and the study population, enhancing the applicability of the findings (188). To assure fair and equitable development of this emerging field, such ethical considerations must be prioritized including as they relate to sharing of data and resources as well as the informed consent process, particularly when vulnerable populations (i.e. those in LICs) are involved.

The generalizability of gut microbiome research can also be improved by the inclusion standardization, longitudinal study designs, and multi-omics analysis. Standardizing methodologies ensures the comparability and reproducibility of gut microbiome research findings (189, 190). To establish guidelines for data generation, processing, and analysis and facilite harmonization across studies, the International Human Microbiome Standards project was established (173, 186); this data will lay the foundation for personalized and innovative methods of prevention, treatment, and management of disease. Longitudinal studies are necessary to understand how the gut microbiome evolves over time and how this relates to health outcomes and/or the pathogenesis of disease. Further, such long-term studies are likely to elucidate unknown relationships between alterations of the gut microbiome and disease development that cannot be studies through observational or cross-sectional studies. Finally, integrating multi-omics data (e.g. genomics, epigenomics, metagenomics, metabolomics) will provide a wholistic understanding of the interactions among the microbiota-gut-brain-immune interface, revealing novel biomarkers and therapeutic targets for numerous disease states. The combination of these will advance personalized healthcare globally.

## Author contributions

AW: Conceptualization, Project administration, Writing – original draft, Writing – review & editing. YN: Conceptualization, Project administration, Writing – original draft, Writing – review & editing. NZ: Writing – original draft, Writing – review & editing. AM: Writing – review & editing. LF: Conceptualization, Project administration, Resources, Supervision, Writing – original draft, Writing – review & editing.

## Supplementary material

Supplementary Table 1 Observed functional relationship with the gut microbiota.
